# Effects of exercise interventions on negative emotions, cognitive performance and drug craving in methamphetamine addiction

**DOI:** 10.3389/fpsyt.2024.1402533

**Published:** 2024-05-17

**Authors:** Conghui Xu, Zunyue Zhang, Dezhi Hou, Guangqing Wang, Congbin Li, Xingfeng Ma, Kunhua Wang, Huayou Luo, Mei Zhu

**Affiliations:** ^1^ School of Medicine, Yunnan University, Kunming, China; ^2^ Yunnan Technological Innovation Centre of Drug Addiction Medicine, Yunnan University, Kunming, China; ^3^ Department of General Surgery I, First People’s Hospital of Yunnan Province, Kunming, China; ^4^ Department of Rehabilitation Education and Corrections, Drug Rehabilitation Administration of Yunnan Province, Kunming, China; ^5^ Department of Gastrointestinal Surgery, The First Affiliated Hospital of Kunming Medical University, Kunming, China; ^6^ Department of Ultrasound, The First Affiliated Hospital of Kunming Medical University, Kunming, China

**Keywords:** methamphetamine use disorder, physical exercise, depression, anxiety, cognition, drug craving

## Abstract

**Introduction:**

Methamphetamine is currently one of the most commonly used addictive substances with strong addiction and a high relapse rate. This systematic review aims to examine the effectiveness of physical activity in improving negative emotions, cognitive impairment, and drug craving in people with methamphetamine use disorder (MUD).

**Methods:**

A total of 17 studies out of 133 found from Embase and PubMed were identified, reporting results from 1836 participants from MUD populations. Original research using clearly described physical activity as interventions and reporting quantifiable outcomes of negative mood, cognitive function and drug craving level in people with MUD were eligible for inclusion. We included prospective studies, randomized controlled trials, or intervention studies, focusing on the neurological effects of physical activity on MUD.

**Results:**

Taken together, the available clinical evidence showed that physical activity-based interventions may be effective in managing MUD-related withdrawal symptoms.

**Discussion:**

Physical exercise may improve drug rehabilitation efficiency by improving negative emotions, cognitive behaviors, and drug cravings.

**Systematic review registration:**

https://www.crd.york.ac.uk/PROSPERO/, identifier CRD42024530359.

## 1 Introduction

Methamphetamine, the main drug in amphetamine-type stimulants, is highly addictive (1). Methamphetamine’s metabolism in the human body is relatively stable. So it soon became one of the most used drugs in the world (1). The latest World Drug Report from the United Nations Office on Drugs and Crime indicates that an estimated 36 million people used amphetamines in 2021, representing 0.7 percent of the global population (2). Qualitative assessments suggest an increase in the use of amphetamines in 2021 and over the last decade (2). In a cross-sectional study of 195 711 respondents in the United States from 2015 to 2019, it was found that methamphetamine use and overdose deaths due to MUD and psychostimulants were on the rise (3).

MUD can lead to many serious consequences, such as anxiety, depression, cognitive impairment, and anhedonia could occur after withdrawal (1). A large proportion of methamphetamine-dependent patients are repeatedly hospitalized and repeatedly detoxified due to MUD-related psychosis (4).In patients with a positive psychiatric history, drug dependence is more likely to lead to long-term psychiatric consequences (5). Therefore, there is an urgent need for a safe, feasible, and effective way to manage methamphetamine addiction.

Exercise is an important part of a healthy life (6), which is conducive to enhancing cardiopulmonary function, improves the functional status of the blood circulatory system (7), and helps to prevent and treat a variety of metabolic diseases, and enhances cognitive ability (8–10). Even low-intensity exercise such as walking is associated with better health (11). In recent years, many studies have confirmed that physical exercise can effectively suppress drug craving and relapse through multiple modulations of the nervous system, and can improve cognitive impairment (12). Furthermore, exercise intervention can help to recover the brain damage (13), facilitate long-term synaptic potentiation-related pathways (14), and promote recovery from severe mental disorders, which was confirmed by real-world multicenter study (15). Exercise has been found to be an effective intervention in improving social health and mental health in people with MUD (16). Moreover, exercise can promote the release of endorphins in the brain, which are naturally occurring analgesics that help to alleviate stress and depression symptoms (17, 18). Physical exercise can also increase the levels of growth factors in the brain, enhancing cognitive function and brain plasticity (19). Results from multicenter randomized controlled trials demonstrated that 20-min moderate physical activity can improve adherence to pharmacological treatments in patients with severe mental disorders (20).

This study aimed to systematically review and synthesize the literature on the efficacy of physical exercise intervention on negative emotion, cognitive impairment, and drug craving caused by MUD. In this systematic review, we provided an overview of the neurological and behavioral effects of exercise interventions as adjunctive treatments for the prevention and elimination of methamphetamine addiction.

## 2 Methodology

Following the Preferred Reporting Items for Systematic Reviews and Meta-Analyzes (PRISMA) 2020 statement (21), this review was registered with the International Prospective Register of Systematic Reviews (PROSPERO CRD42024530359).

To summarize recent findings from the effects of exercise on the management of methamphetamine addiction, we searched the PubMed and Embase databases for articles published in peer-reviewed journals written in English and indexed until October 2023. We used search line such as (((physical exercise) OR (physical activity) OR (exercise)) AND((methamphetamine) OR (methamphetamine use disorder))) AND((depression) OR (anxiety) OR (cognition)). The search strategy was limited to the period from 2014 to 2023. All titles and abstracts found by the search strategy as reported were screened for relevance by GQW, CBL, and XFM. Full searches of titles and abstracts were then conducted to assess eligibility for inclusion in the review by DZH and GQW, independently.

The following eligibility criteria have been considered: published in English; participants met diagnostic criteria for MUD according to the fifth edition of the American Psychiatric Association’s Diagnostic and Statistical Manual of Mental Disorder (DSM-5); used clearly described physical activity as interventions; outcomes included negative mood, cognitive function, or drug craving levels with quantifiable indicators. Studies with insufficient evidence or very low quality, studies not focusing on MUD, and studies in animal models or *in vitro* were excluded. Non-original research, such as conference papers, systematic reviews, meta-analyses, and narrative reviews were excluded.

**Figure 1:**
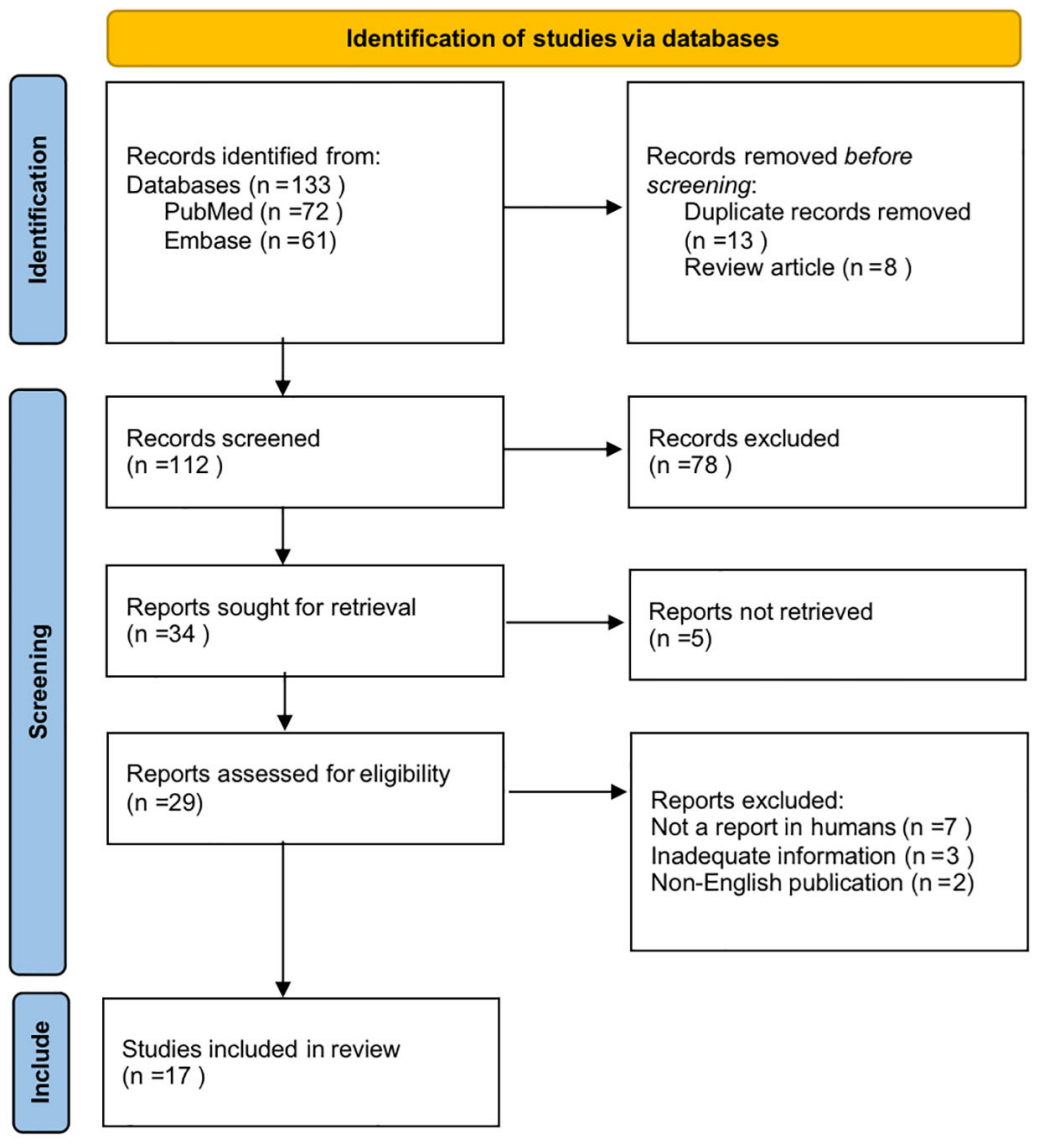
The flow chart showing the Methods and strategies of the review.

Based on the above criteria, all results were in independently selected for eligibility by CHX, and ZYZ, with disagreements resolved by discussion. CHX and ZYZ independently extracted the data regarding sample characteristics, intervention, duration, intensity, outcome measures, and outcomes for each selected study. HYL, MZ and KHW supervised the entire process. A total of 17 studies out of 133 were included in this review. The detailed process is shown in **Figure 1**. Characteristics of all studies included in the review were described in **Table 1**. We included prospective studies, randomized controlled trials, or intervention studies, focusing on the neurological effects of physical activity on MUD. The risk of bias was assessed by two independent reviewers using the AMSTAR2 tool (38).The purpose of this review is to provide an overall assessment discussion of the current state of research. We did not request additional data from authors of published reports, nor did we analyze data not included in the reported articles selected in the systematic review.

**Table 1:** Characteristics of all studies included.

Ref	Sample	Intervention	Duration	Intensity	Outcome measures	Outcomes
(16) 2022	N=60 exercise group mean age=31.3	aerobic exercise	five days a week for 60 min each for 12 weeks	moderate-intensity	quality of life scale for drug addiction (QOL-DA), self-rating anxiety scale (SAS), self-rating depression scale (SDS), and Pittsburgh sleep quality index (PSQI) physical fitness	social health improved; mental health improved; physical health improved
(22) 2015	N=135 mean age=31.7	aerobic resistance training	60-minute sessions, three times a week over 8 weeks	individually tailored and progressive	Beck Depression Inventory questionnaire MINI International Neuropsychiatric Interview	significant effect of exercise on reducing depression and anxiety
(23) 2015	N=135 exercise group mean age=31.9	aerobic and resistance exercise	3-times-per-week, 60-minute for 8 weeks	progressive structured exercise program	Beck Depression Inventory Beck Anxiety Inventory	reduce depression and anxiety symptoms
(24) 2021	N=36 female only mean age=26.89	acute exercise (dancing or stationary cycling)	30 mins	moderate-intensity	neural activity in prefrontal lobe structures by functional near-infrared spectroscopy (fNIRS) Self-Rated Emotional Assessment	ameliorate negative emotional reactions by decreasing attention to negative stimuli
(25) 2016	N=92 aged 18–40 years	aerobic exercise	30 mins	light/moderate/high	visual analog scale (VAS) Go/No Go task event-related potentials (ERPs) time-locked electroencephalogram (EEG)	moderate-intensity exercise have larger effects on drug craving and inhibitory control
(26) 2021	N=466 male only exercise group mean age=31.27	aerobic exercise	five 60 min sessions a week for 8 weeks	moderate-intensity	cardiopulmonary fitness, HRV time-domain, frequency-domain parameters, executive function(the Stroop task, the 2-back, and the Shift-task)	autonomic nervous system function and executive function improved.
(27) 2021	N=98 female only exercise group mean age=29.93	aerobic exercise	40 mins,5 times every week for 3 months	moderate-intensity	Attention and working memory (Trail Making Test and Digit Span Test), verbal memory (Logical Memory (LM) and Memory for Persons Data (MPD)), executive function (Color-Word Stroop Test)	attention, working memory, executive function, and parts of verbal memory improved
(28) 2022	N=47 male only HIIT group mean age=32.7	high-intensity interval training (running, resistance training, rope skipping) or Tai chi	1h, three times a week for 6 mouths	high-intensity	computer-based Go/No-go and Stroop tasks	inhibitory control promoted; HIIT group showed greater improvements in response accuracy
(29) 2021	N=240 Age=18–40	high-intensity interval training(treadmill) moderate-intensity continuous training (combine TC, Qigong and Yoga)	24 mins, three times a week for 6 mouths	high/ moderate	Stroop test; Wechsler Adult Intelligence Scale-III; Cognitive function test; Craving Automated Scale for Substances (CAS-S);Mental health characteristics	HIIT training reduce illicit drug cravings and have a good effect on the cognitive functions
(30) 2015	N=24 mean age=31.46	acute stationary cycle exercise	30 mins	moderate intensity	VAS Go/No go paradigms ERP	beneficial effects on drug craving and inhibitory control
(31) 2018	N=68 patinets mean age=33.29	aerobic exercise (cycling, jogging, or jump rope)	three 30 min sessions per week for 12 weeks	moderate-intensity	CogState battery, international shopping list task (ISL), detection task (DET), identification task (IDN), one card learning task (OCL), two back task (TWOB), social emotional cognition task (SEC), continuous paired association learning task (CPAL), and Groton maze learning task (GML))	aerobic exercise program may have beneficial effects on the processing speed as well as blood lipid peroxidation in patients with MUD
(32) 2021	N=30 mean age=32.3	VR competitive cycling	10 mins	high intensity	color-word Stroop task profile of mood states (POMS) fNIRS	facilitates executive information processing by enhancing task-related cortical activations and brain functional network efficiency
(33) 2021	N=32 mean age=42.34	Tai chi	45 mins, three times a week for four weeks	moderate-intensity	drug-related Stroop task	general inhibition control enhanced
(34) 2021	N=36 female only mean age=26.8	dance or treadmill	35 mins	moderate-intensity	functional near-infrared spectroscopy (fNIRS) visual analog scale (VAS) electronic appetite self-rating system	cue-induced drug craving decreased; food reward and appetite improved
(35) 2021	N=90 male only exercise group mean age=32.75	Aerobic combined resistance exercise	60 min, five times a week, practice 8 weeks	structured and progressive	plasma IL-6, TNF-α, and IL-1β concentrations; VAS; Desires for Drug Questionnaire (DDQ)	peripheral inflammation and the level of craving for methamphetamine significantly reduced
(36) 2022	N=112 female only mean age=32.54	Tai Chi	3 mouths	moderate-intensity	cue-induced drug craving assessment VAS	craving score reduced
(37) 2015	N=135 exercise group mean age=31.9	treadmill	55 mins, 3 times a week for 8 weeks	moderate-intensity	methamphetamine use as measured by both urine drug screens (UDS) and self-report using the Substance Use Inventory	resulted in a decrease in METH use among lower severity METH users at 1-month, 3-months, and 6-months post treatment

## 3 Results

### 3.1 Negative emotions

Anxiety and/or depression is the most common mental symptom in the process of methamphetamine use and withdrawal, more than 40% of users have varying degrees of anxiety and/or depression, which directly affects the success rate and relapse rate of withdrawal (39). Effectively improving the negative emotions of patients is one of the key medical problems to be solved urgently in drug treatment (40). Methamphetamine binds to dopamine, norepinephrine, and serotonin transporters in neurons, leading to the rapid accumulation of monoamine neurotransmitters in brain synapses (1). MUD affects the brain’s reward pathway by releasing these neurotransmitters to induce euphoria (41, 42). Long-term abuse of methamphetamine can severely impair the structure and function of the monoamine transmitter system in the brain of methamphetamine users, leading to depletion of the monoamine neurotransmitter (43) and disturbance in the release of neurotransmitters such as dopamine (DA), norepinephrine (NE), and serotonin (5-HT) (1, 44). Besides, long-term abuse of methamphetamine depletes dopamine reserves in the brain and reduces the availability of dopamine receptors (45). Changes in dopamine levels in the brain can regulate emotional states such as depression and anxiety, resulting in reward effects, drug cravings, etc. (1). In addition, 5-HT and NE neurotransmitters can also modulate synaptic plasticity and improve negative mood, which is strongly associated with methamphetamine dependence (46).

Methamphetamine also impairs movement, executive performance, and episodic memory, leading to anxiety and depression (47). Positron emission tomography imaging has found that dopamine transporters in the caudate/putamen and nucleus accumbens of methamphetamine users are significantly reduced, and are significantly correlated with the duration of methamphetamine use and the severity of persistent psychiatric symptoms (48). A positive correlation between methamphetamine dependence severity and incidence of depression and anxiety has also been demonstrated in clinical populations (49). Methamphetamine withdrawal syndrome presents with behavioral psychiatric symptoms such as drug cravings, lack of pleasure, irritability, difficulty concentrating, lethargy, and even suicide (50, 51). Besides, negative emotions like depression and anxiety are also strongly associated with relapse and rehabilitation in methamphetamine-dependent individuals in the early stages of rehabilitation withdrawal (52, 53). Studies have shown that physical activity can improve physiological and neurological damage caused by methamphetamine dependence, increase the release of brain-derived neurotrophic factor (BDNF), and exert antidepressant-like effects (54).

In people with mood disorders, aerobic exercise interventions for 8 to 12 weeks have shown some therapeutic efficacy and significant improvement in negative mood (55). A study of 60 methamphetamine-dependent individuals showed that 12 weeks of moderate-intensity aerobic exercise significantly improved the patients’ social health, mental health, and physical health (16). Some researchers found that after 8 weeks of moderate-intensity exercise, participants who received exercise interventions had significantly lower depression scores than healthy groups, while participants who received the most frequent exercise had the best results. It was further concluded that exercise interventions are more effective in the early stages of addiction withdrawal and can be effective in treating depressive symptoms in individuals (22). Researchers found that an eight-week exercise program significantly improved depression and anxiety symptoms in patients with MUD during the acute withdrawal phase, with significantly lower levels of depression and anxiety in the exercise group than in the healthy control group during the eight-week follow-up period (23). A 30-minute dance session can also improve negativity by reducing the attention of female methamphetamine dependents to negative stimuli (24).

The therapeutic effect of exercise also has been confirmed in animal experiments. For example, wheeled running can reduce central and peripheral inflammation and relieve anxiety-like symptoms in mice with an acute withdrawal model of methamphetamine (56).

### 3.2 Cognitive performance

Chronic drug abuse is often accompanied by a decrease in an individual’s ability to suppress control, and deficits in inhibitory control in cognitive control are also an important marker of drug dependence (57, 58). In different types of drug users, inhibition control of stimulant users (e.g., cocaine, methamphetamine, etc.) exhibited a higher response error rate, longer response time, and lower inhibition compared with opioid users (59). Studies have found that patients with MUD are often accompanied by abnormal cortical striatal function such as abnormal gray matter and white matter integrity, monoamine neurotransmitter system defects, neuroinflammation, and poor neuronal integrity (1, 60). These neurological abnormalities can seriously affect individual cognitive function (61, 62). Changes in neuroplasticity and the remodeling of specific brain circuits may be the primary neural basis for the effects of drug exposure on learning and memory (63–65).

Hence, patients with methamphetamine addiction often exhibit impaired cognitive function, including executive function, attention, social cognition, flexibility, and working memory (66, 67). Memory is also one of the important aspects of the cognitive abilities. Long-term abuse of methamphetamine may eventually affect learning and memory by altering the neuroplasticity of associated brain regions, such as the dorsal hippocampus (41) and prefrontal cortex (68). Evidence from animal models showed that the synaptic transmission rate in the dorsal hippocampus may be reduced by exposure to methamphetamine during adolescence, ultimately contributing to the development of memory impairment (64, 68, 69).

Physical activity has been shown to improve cognition in cognitively impaired populations, and the therapeutic effect of exercise intensity varies (70–72). In studies of methamphetamine users, experimental results have shown that moderate-intensity aerobic exercise has the most significant effect on inhibition and control (25). A randomized controlled trial of 330 people with MUD has validated the efficacy of 8 weeks of aerobic exercise and showed that aerobic exercise can improve autonomic nervous system function and executive function in individuals with MUD at the same time (26). Randomized controlled trials have also demonstrated that 12 weeks of aerobic exercise can improve attention, working memory, and executive function in women with methamphetamine dependence, and promote cognitive recovery (27). High-intensity intermittent aerobic exercise has also been found to improve the executive ability and response accuracy of methamphetamine-dependent patients (28, 29). Moreover, there are differences in the effects of short-term acute exercise and long-term adaptive exercise on inhibition capacity. Behavioral and electrophysiological measurements were performed by the Go/No Go association task in the absence of pharmacological intervention to examine the effect of acute exercise on craving-related craving and inhibitory control in methamphetamine users, which showed that acute aerobic exercise can increase inhibition (30). The therapeutic effect of chronic aerobic exercise on inhibition has also been reported. For example, MUD patients were given 12 weeks of moderate-intensity aerobic exercise training and found significant improvements in lipid peroxides and cognitive function (31). In addition to this, there have also been studies that have reported the efficacy of various forms of exercise. Acute VR competitive cycling can enhance information processing capacity by enhancing task-related cortical activation and brain functional networking in methamphetamine-dependent individuals (32).

Tai Chi is a traditional Chinese sport that is classified as a moderate-intensity exercise. Attention bias towards medication is an important indicator of MUD, and 4 weeks of tai chi exercise reduced the sensitivity and attention bias of medication-related cues in people with MUD, suggesting that tai chi exercise intervention may promote recovery from MUD through attention control (33).

### 3.3 Drug cravings

The potentiating effect of the drug depends primarily on dopamine signaling in the nucleus accumbens, and long-term drug exposure triggers glutamate-mediated neuroadaptation in the dopamine striatum-thalamic cortex (prefrontal cortex regions, including the orbitofrontal and anterior cingulate cortex) (60) and marginal pathways (amygdala and hippocampus), which can lead to addiction (73). Drug cravings are a key factor in maintaining drug dependence. methamphetamine users have more severe drug cravings and anxiety than heroin users (74, 75). Long-term drug abuse can induce the release of γ-aminobutyric acid and glutamate and the differential expression of DA neurons in the ventral tegmental region of the midbrain, leading to addiction and perpetuation of drug use (76–78). Some studies have also shown that homeostatic imbalance of midbrain dopamine caused by drug addiction is associated with decreased levels of dopamine D2 receptors in the striatum (42, 79). Numerous studies using positron tomography have demonstrated that the availability of dopamine D2/D3 receptors in the striatum is associated with drug cravings for methamphetamine-addicted (80, 81) and mediates impulsive temperament personality (82).

As a non-pharmacological treatment, exercise can activate the reward system, which in turn affects the neural circuits and neurotransmitter conduction of drug users, so exercise may reduce drug-induced dependent behaviors (17, 18, 83, 84). In the reward pathway of drug addiction, physical activity can enhance the ability of dopamine signaling to reduce drug overuse. In population experiments on methamphetamine addiction, subjects in the exercise group showed significant increases in striatal D2/D3 receptor availability (measured as non-displaceable binding potential (BPND)) (85). Furthermore, study have shown that wheeled running can improve methamphetamine-induced dopamine and serotonin homeostasis imbalances and regulate the normalization of reward pathways (86). Therefore, physical exercise may improve dopamine system function by promoting an increase in dopamine release and increasing the expression of dopamine D2 receptors, thereby improving brain reward pathways, reducing the drug craving (85).

A study reported that acute moderate-intensity dance and aerobic exercise may decrease cue-induced methamphetamine craving and improve food reward and appetite responses in women with MUD (34). 8 weeks of aerobic exercise combined with resistance training reduced levels of inflammatory factors in the peripheral blood and significantly reduced the craving for methamphetamine in people with MUD (35). A study in people dependent on methamphetamine showed that drug craving level decreased significantly during exercise, immediately after exercise, and 50 minutes after exercise, and drug craving scores at these time points after exercise were significantly lower than in the control group. In studies of MUD patients, 20 minutes of moderate-intensity acute aerobic exercise was found to temporarily relieve cravings in those with methamphetamine withdrawal (30). In a follow-up study, the investigators compared the dose-response relationship between different exercise intensities (low, moderate, and high intensity) and methamphetamine cravings. Furthermore, randomized controlled trials have demonstrated that tai chi can help reduce withdrawal symptoms by increasing individual self-awareness and introverted attention, and also have a good therapeutic effect on drug cravings in methamphetamine-dependent patients (36). In terms of intensity, results showed that methamphetamine craving scores decreased more in the moderate- and high-intensity exercise groups than in the low-intensity exercise and control groups during acute exercise, immediately after exercise, and at 50 minutes after exercise (25). Additionally, researchers found a significant reduction in the use of methamphetamine after eight weeks of aerobic exercise intervention (37).

Studies in animal models have shown that six weeks of treadmill exercise upregulate levels of dopamine D2 receptors in the striatum and regulate dopamine homeostasis in the midbrain (87).Furthermore, in methamphetamine-dependent rats, a reduction in methamphetamine craving induced by chronic wheel running is associated with the number of gray matter dopamine neurons around the midbrain aqueduct (88).

## 4 Discussion

The substance use disorder is a long-standing world problem. The increasing complexity of global drug abuse in the wake of the pandemic, and the inevitable cultivation of illicit drug plants triggered by the economic downturn, have given rise to flexible drug delivery models (89). Drug rehabilitation is a difficult and necessary task. Together, the results from this systematic review suggest there is consistent evidence that exercise therapy may play a positive role in drug rehabilitation through a variety of neurobiological mechanisms to regulate the physiological function and psychological state of people with MUD.

We included 17 studies within the last decade. The majority of included studies adopted randomized controlled design. The sample size ranged from 24 to 466 with a total of 1836 participants. The age of these participants ranged from 18 to 45 years old, with the average age mostly around 31 years old. They did not receive any specific treatment other than exercise interventions. The exercise group and controls were matched for age, education. Of these studies, exercise interventions include treadmill, tai chi, resistance exercises, cycling, and dance, with varying intensity and duration. The control group was given health education by watching relevant materials or videos.

In terms of intervention intensity, moderate-intensity exercise (16, 24–27, 30, 31, 34, 37)and high-intensity interval training (28, 29, 32) have been shown to have greater benefits. However, due to experimental constraints, these studies have been inconsistent on the duration of exercise, ranging from short-term acute aerobic exercise (24, 25, 30, 32, 34) to sustained exercise lasting weeks or months (16, 22, 23, 26–29, 31, 33, 35–37). Analyzing the effects of different intervention duration and intensity on people with MUD is conducive to the subsequent formulation of a unified exercise intervention treatment plan.

These studies confirmed that exercise interventions can reduce anxiety and depression symptoms (16, 22–24), enhance inhibitory control (25, 28–30, 33) and executive capacity (26, 27), and reduce the level of craving for methamphetamine (25, 30, 34–37) in people with MUD. Although further research on the efficacy of exercise interventions is required, based on the available evidence, we assume that exercise interventions have some advantages in the treatment of MUD.

The role of exercise in MUD is well reported. However, significant problems and challenges still remain, hindering the large-scale adoption of exercise interventions, that future research should address to better understand its clinical implications. Firstly, differences in the role of different exercise patterns, exercise frequency and duration in suppressing addictive behaviors are not exactly clear. Secondly, no efficient, sensitive and objective biomarkers have been found, the possibility of BDNF and proBNDF as biomarkers to predicate disease stage, synaptic plasticity, and therapeutic efficacy could be considered in the future (90, 91). Finally, patients with different states need a normative guideline for exercise interventions. To address these issues, further analyses of the neurobiological mechanisms of physical activity interventions are necessary.

In particular, more specific neural network mechanisms, and more *in vivo* evidence of the addiction model are needed to provide more theoretical support. Besides, preclinical studies and clinical trials could combine physical activity with some promising treatments or medicaments, such as repetitive transcranial magnetic stimulation (92) or bupropion, mirtazapine, and methylphenidate (93).Psychosocial and psychological interventions have also been proved to be effective in patients with mood disorders (94). Physical activity may improve the efficacy of these promising therapy. Moreover, there are many types of drugs except methamphetamine. The role of physical exercise in patients who use different types of drugs, as well as the development of personalized exercise prescriptions, are also the directions of further research and analysis.

Additionally, many forms of exercise can be used as effective intervention methods in drug treatment, such as mindfulness meditation (95, 96), traditional Chinese health exercises (28, 33, 36, 97)(e.g. Health Qigong, Tai Chi), and other forms of exercise that take into account both physical and psychological aspects. The practice of those therapies requires the elimination of distractions, concentration, and a calm mind. This is actually a process of psychological adjustment, allowing people in forced rehabilitation to regain their concentration, strengthen their inner self-awareness, and then get rid of negative emotions. Furthermore, traditional health-preserving exercises are mostly group activities and group communication activities, which can promote people’s socialization and interpersonal relationship development, thereby regulating negative emotions and enhancing happiness. More evidence-based medical evidence is needed on these forms of exercise.

## 5 Conclusion

In summary, exercise could be used as a safe, accessible, and effective intervention to alleviate the mental health problems associated with MUD. It is anticipated that continued research on the therapeutic role of physical exercise interventions will aid in the development of effective drug rehabilitation programs for the treatment of drug-dependent individuals. However, there is a need to collect more high-quality evidences all over the world, and the efficacy and sustainability of physical activity interventions should be evaluated in more longer-term studies.

## 6 Limitations of the study

This systematic review presents several limitations: The number of studies included in this review was limited. Moreover, due to study constraints, the gender distribution of study participants, the modalities of exercise interventions, and the interventions in the control group were also inconsistent. Most of these studies came from China due to its compulsory drug rehabilitation institutions.

## Data availability statement

The original contributions presented in the study are included in the article/supplementary material. Further inquiries can be directed to the corresponding author/s.

## Author contributions

CX: Writing – original draft, Writing – review & editing. ZZ: Writing – review & editing. DH: Writing – review & editing. GW: Writing – review & editing. CL: Writing – review & editing. XM: Writing – review & editing. KW: Funding acquisition, Writing – original draft, Writing – review & editing. HL: Funding acquisition, Writing – original draft, Writing – review & editing. MZ: Writing – original draft, Writing – review & editing.
